# Successful surgical management of mesenteric inflammatory veno-occlusive disease

**DOI:** 10.1186/s40792-020-0796-1

**Published:** 2020-01-21

**Authors:** Keiji Matsuda, Yojiro Hashiguchi, Yoshinao Kikuchi, Kentaro Asako, Kohei Ohno, Yuka Okada, Takahiro Yagi, Mitsuo Tsukamoto, Yoshihisa Fukushima, Ryu Shimada, Tsuyoshi Ozawa, Tamuro Hayama, Takeshi Tsuchiya, Keijiro Nozawa, Yuko Sasajima, Fukuo Kondo

**Affiliations:** 1 Department of Surgery, Teikyo University School of Medicine, 2-11-1 Kaga, Itabashi-ku, Tokyo, Japan; 2 Department of Pathology, Teikyo University School of Medicine, Tokyo, Japan

**Keywords:** Mesenteric inflammatory veno-occlusive disorder, Surgery, Stenosis, Bleeding

## Abstract

**Background:**

The term “mesenteric inflammatory veno-occlusive disease (MIVOD)” is used to describe an ischemic injury resulting from phlebitis or venulitis that affects the bowel or mesentery in the absence of arteritis. MIVOD is difficult to diagnose because of its rarity and frequent confusion with other diseases. The incidence and etiology of MIVOD remain unclear; only a few cases have been reported. We describe a case of the successful surgical management of a patient with MIVOD with characteristic images.

**Case presentation:**

A 65-year-old Japanese man visited a hospital with the chief complaint of abdominal pain in January 2018. CT showed edema and thickening of the intestinal wall from the descending colon to the rectum. The patient was admitted to the hospital. Suspected diagnoses were enteritis, ulcerative colitis, amyloidosis, vasculitis, malignant lymphoma, and venous thrombus, but no definitive diagnosis was obtained. The patient was transferred to our hospital for the treatment of stenosis (located from the descending colon to the rectum) and bowel obstruction. An emergency transverse colostomy was performed. The sigmoid colon and mesentery were too rigid and edematous to resect. Colonic hemorrhage occurred 2 weeks after the surgery. With radiology intervention, coiling for the arteriovenous fistula in the descending colon was performed, and hemostasis was obtained. A colonoscopy at 6 months post-surgery showed neither ulceration nor stenosis in the rectum, indicating that the rectum could be preserved in the next surgery. However, severe stenosis in the descending and sigmoid colon remained unchanged. Ten months after the transverse colostomy, we performed a subtotal colectomy and ileorectal anastomosis, and an ileostomy was created. The sigmoid colon and mesentery were not so rigid compared to the first surgery’s findings, and we were able to resect intestine and mesentery. Histopathology revealed phlebitis and venulitis, fibrinoid necrosis, and normal arteries, meeting the diagnostic criteria for MIVOD. Postoperatively, the patient showed no recurrence for 8 months.

**Conclusion:**

Clinicians should consider MIVOD when examining a patient with intestinal ischemia. When MIVOD is suspected, the patient is indicated for surgery based on an accurate diagnosis and good prognosis.

## Background

Mesenteric inflammatory veno-occlusive disease (MIVOD), a rare cause of inflammatory enterocolitis, has clinical and imaging features that can be confused with those of mesenteric venous thrombosis or inflammatory bowel disease (IBD) [1, 2]. MIVOD is used to describe an ischemic injury resulting from phlebitis or venulitis that affects the bowel or mesentery in the absence of arteritis [1, 3]. Because of its rarity and frequent confusion with other diseases, MIVOD is difficult to diagnose [4]. MIVOD is also resistant to medical treatment and anticoagulants, and immunoregulatory drugs have proven ineffective for patients with MIVOD [5]. The incidence and etiology of MIVOD remain unclear in part because only a few cases have been reported. We describe a case of the successful surgical management of an individual with MIVOD with characteristic images.

## Case presentation

**Fig. 1:**
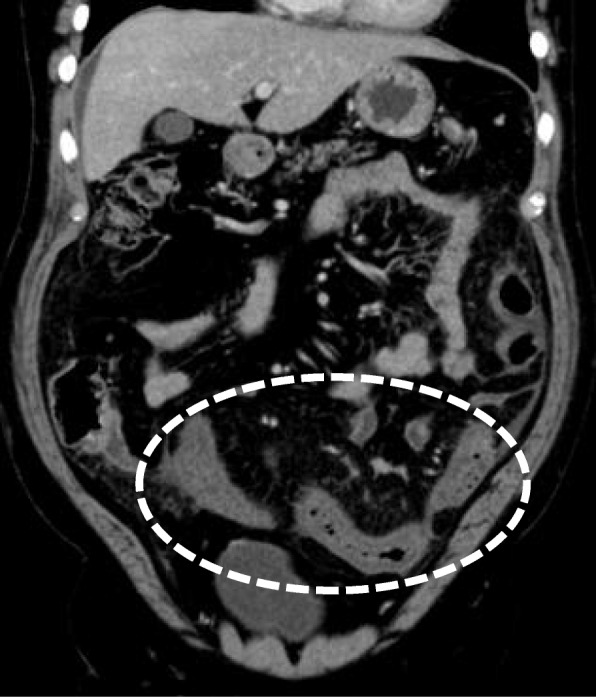
Portal venous-phase CT of the abdomen demonstrating marked wall thickening with poor mural enhancement and extensive submucosal edema in the sigmoid colon (white dotted circle)

**Fig. 2:**
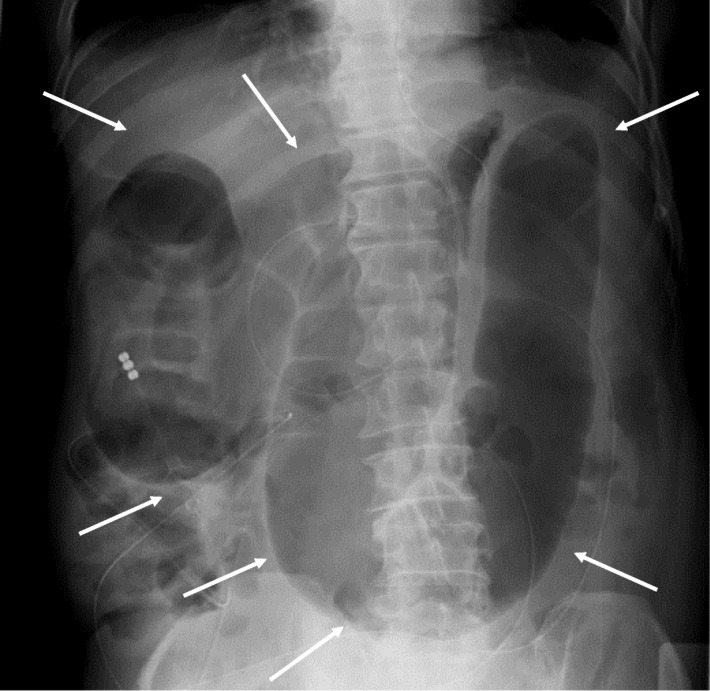
Dilatation of the transverse colon was observed (white arrows)

**Fig. 3:**
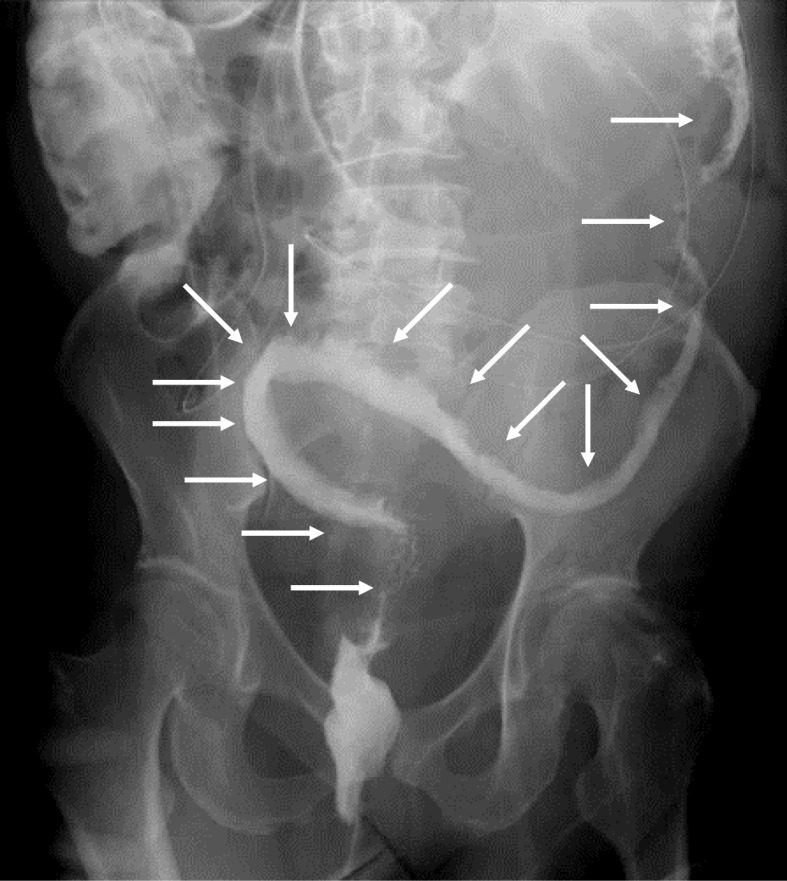
Enema showed narrowing from the descending colon to the rectum (white arrows)

In January 2018, a 65-year-old Japanese man visited a hospital with the chief complaint of abdominal pain. CT showed edema and thickening of the intestinal wall from the descending colon to the rectum (Fig. 1). The blood examination results included a white blood cell (WBC) count at 6500/μl and the C-reactive protein (CRP) level at 13 mg/dl. The patient was admitted to that hospital based on the suspicion of enteritis. The result of a fecal culture was negative. Despite fasting and antibiotic treatment, the patient's condition did not improve. The differential diagnoses were ulcerative colitis, amyloidosis, vasculitis, malignant lymphoma, and venous thrombus, but no definitive diagnosis was obtained. On the 48th hospital day, dilatation of ascending colon to transverse colon was observed (Fig. 2). A long tube was inserted through the nostril to treat the intestinal obstruction. Strong narrowing of the descending colon to rectum was revealed by an enema X-ray examination (Fig. 3).

**Fig. 4:**
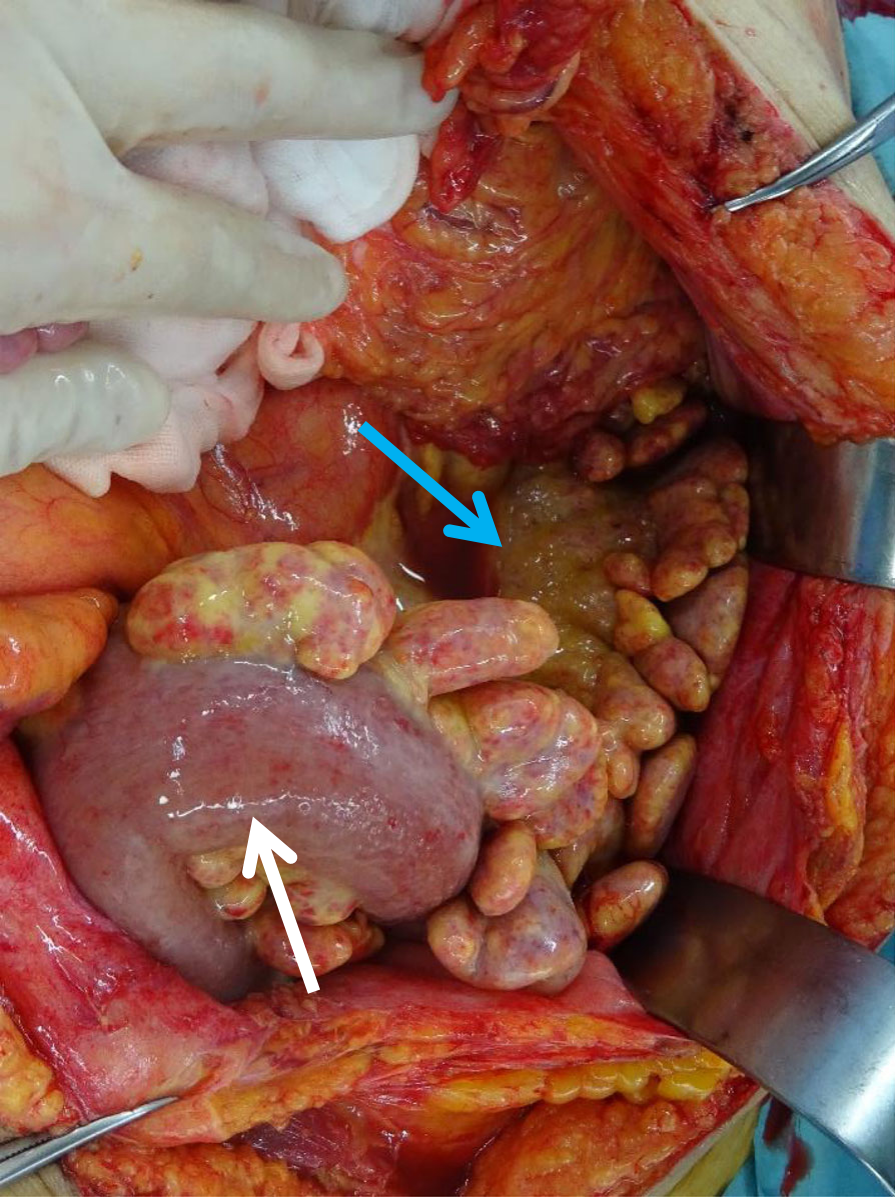
At the first surgery, the sigmoid colon (white arrow) and mesentery (blue arrow) were too rigid and edematous to resect

**Fig. 5:**
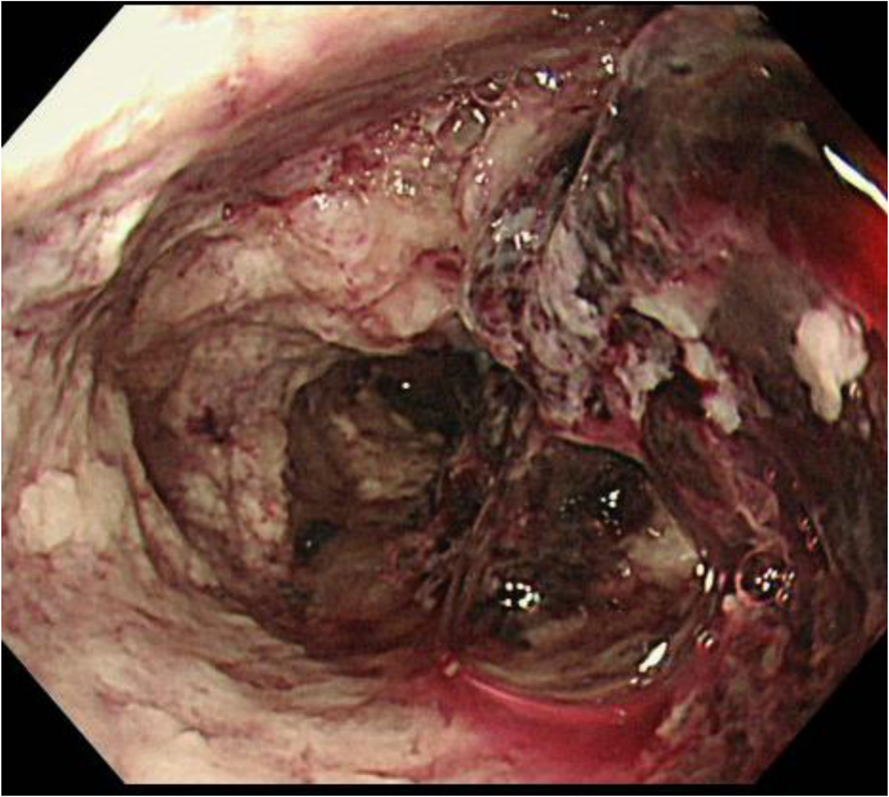
Colonoscopy showing hemorrhagic circumferential ulceration

**Fig. 6:**
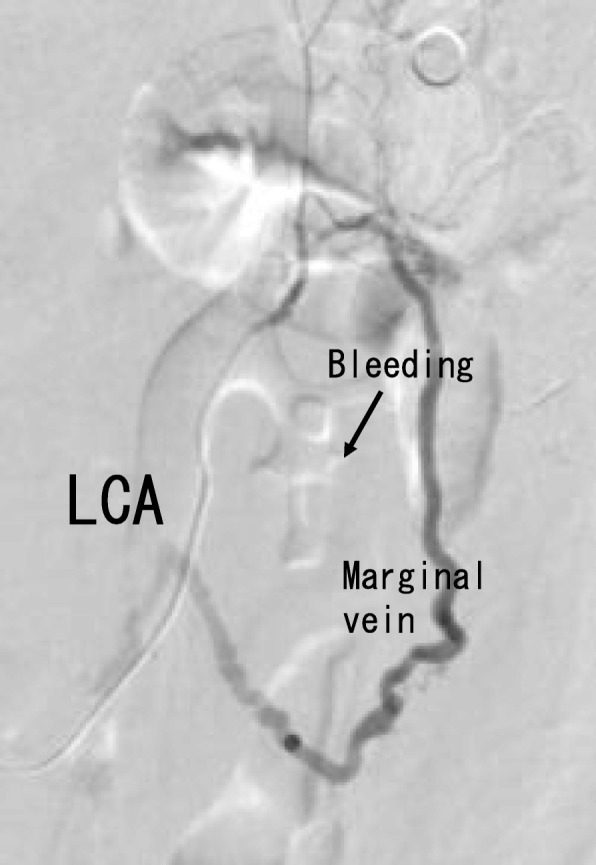
Angiography showing an arteriovenous fistula in the descending colon

On the 57th day of hospitalization, the patient was transferred to our hospital for the treatment of colonic stenosis and bowel obstruction. On the same day, an emergency transverse colostomy was performed. We tried to resect bowel, however, the sigmoid colon and mesentery were too rigid and edematous to resect (Fig. 4). Prednisolone was started with the diagnosis of mesenteric panniculitis. Hemorrhage occurred 2 weeks after the transverse colostomy surgery. Colonoscopy showed circumferential ulceration, and achieving hemostasis by colonoscopy was considered too difficult (Fig. 5). With the intervention of radiology, coiling for the arteriovenous fistula was performed, and hemostasis was obtained (Fig. 6).

**Fig. 7:**
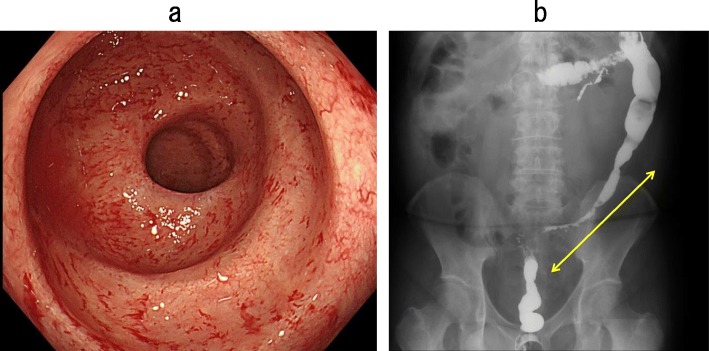
Colonoscopy 6 months after the surgery showed neither ulceration nor stenosis in the rectum (**a**), but an enema X-ray examination showed severe stenosis in the descending and sigmoid colon (**b**, yellow line)

The patient was discharged from our hospital at 51 days after the transverse colostomy surgery. Six months later, colonoscopy showed neither ulceration nor stenosis in the rectum, indicating that the rectum could be preserved in the next surgery (Fig. 7a). However, the severe stenosis in the descending and sigmoid colon remained unchanged (Fig. 7b).

**Fig. 8:**
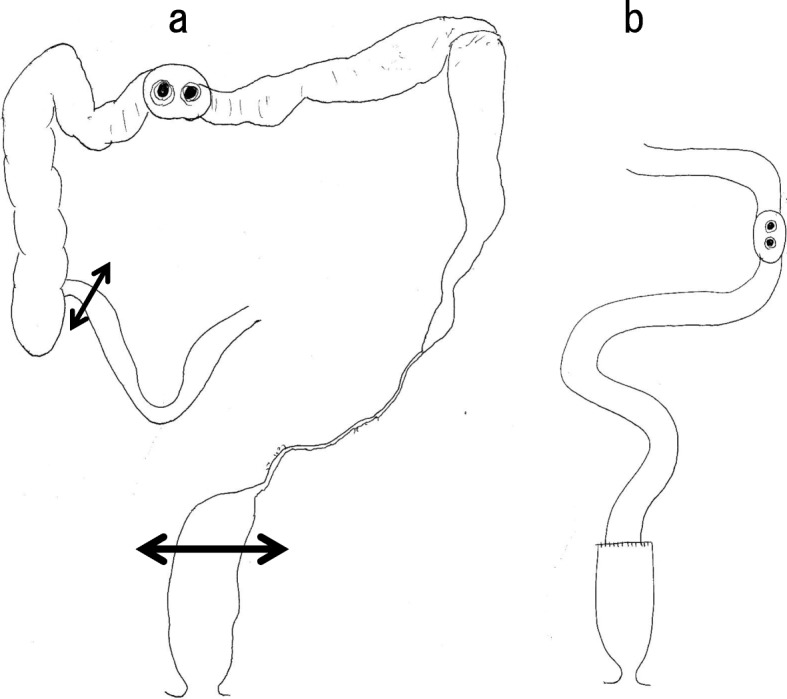
Subtotal colectomy (**a**), ileorectal anastomosis, and ileostomy creation (**b**) were performed

**Fig. 9:**
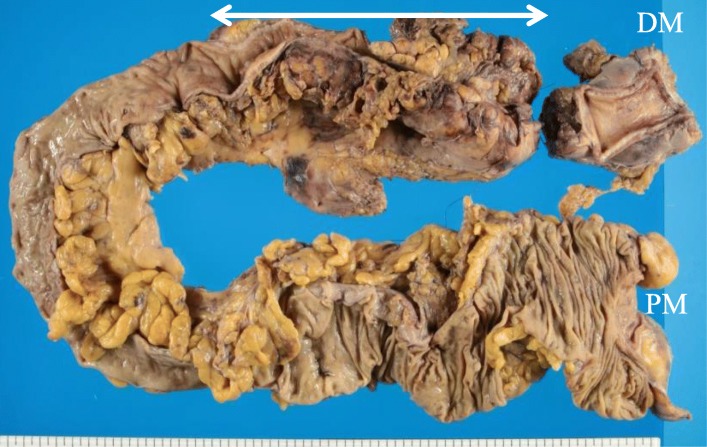
Resected specimen showing severe stenosis from the descending colon to the sigmoid colon (white arrow)

**Fig. 10:**
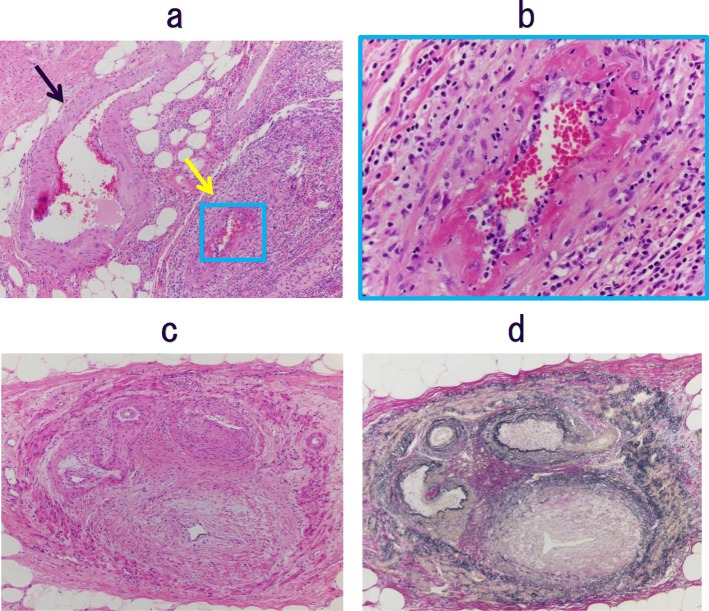
The arteries and veins penetrating through muscularis propirae. Vein showing phlebitis with fibrinoid necrosis, but arteries were normal (**a**, hematoxylin/eosin [HE] stain, black arrow: artery, yellow arrow: vein). Magnified image shows phlebitis with fibrinoid necrosis (**b**, HE stain). A recanalized finding of a thick vein was observed at the subserosal layer (**c**, HE stain). Prominent stenosis was observed in the recanalized vein (**d**, Elastica van Gieson stain)

Ten months after the creation of the transverse colostomy, we performed a subtotal colectomy, ileorectal anastomosis, and ileostomy creation (Fig. 8). The sigmoid colon and mesentery were not so rigid compared to the findings in the first surgery, and we were able to resect intestine and mesentery (Fig. 9). Because the length of the remnant rectum was only 7 cm, it was difficult to anastomose the right colon to the remnant rectum. The histopathology evaluation revealed phlebitis and venulitis, fibrinoid necrosis, and normal arteries (Fig. 10). These findings were diagnostic of MIVOD with myointimal hyperplasia of mesenteric veins. The patient’s postoperative course was uncomplicated. The ileostomy is scheduled to be closed 8 months after the second surgery.

## Discussion

Obliterative venous thrombophlebitis of the intestine is not a common disease; in 1994 Flaherty described it as a new clinicopathologic entity named “mesenteric inflammatory veno-occlusive disease” or MIVOD [1]. The findings of MIVOD were established as follows: (1) phlebitis and venulitis affecting veins of the bowel and mesentery and resulting in ischemic injury of the bowel are present, (2) vasculopathy is the only demonstrable cause of the ischemia, (3) arteritis is not present, and (4) a history of extraintestinal vasculitis is not evident [1, 2].

We searched the following sources for studies reporting cases of MIVOD: Ovid, Embase, and MEDLINE (using PubMed). The search terms included MIVOD in all fields. Our search identified 30 documents, and 33 cases were extracted [1–13]. Table 1 summarizes these past cases and our patient’s case. Together the patients consisted of 22 men and 12 women. The durations of symptoms ranged from 1 day to 4 months. The affected organs were the jejunum (*n* = 10), ileum (*n* = 5), colon (*n* = 16), jejunum and colon (*n* = 1), gallbladder (*n* = 1), and omentum (*n* = 1). Many cases underwent segmental resection; however, subtotal or total colectomy was performed in three patients, no. 23, no. 24, and no. 32 besides our case (Table 1). Segmental resection for the affected lesion had better be attempted, in some cases wide range of bowel must be removed. Thirty of the 33 patients (88%) showed a good prognosis, but one patient (no. 5) died with postoperative complications [1] and the remaining patient (no. 19) died with recurrent mesenteric ischemia [3]. One patient (no. 28) showed recurrent MIVOD but was alive after surgery [8].

**Table 1:** Clinical presentation of the reported patients with MIVOD

No.	Age (years)	Sex	Prodromal symptoms at visit	Comorbidity	Medications	Smoker	Bowel affected	Time to operation from the onset	Operation method	Outcome	References
1	78	Female	3-4 wk	N/D	N/D	N/D	SC	N/D	Segmental resection, OP	NED, 6 mo	[1]
2	34	Male	2-3 wk	N/D	N/D	N/D	Jejunum	N/D	Segmental resection, OP	NED, 7 mo	[1]
3	27	Female	1 wk	N/D	N/D	N/D	Jejunum	N/D	Segmental resection, OP	NED, 7 mo	[1]
4	46	Male	several mo	N/D	N/D	N/D	SC	N/D	Segmental resection, OP	NED, 18 mo	[1]
5	36	Female	2 wk	N/D	N/D	N/D	Right colon	N/D	Segmental resection, OP	Died 1 mo postoperatively, ARDS and DIC	[1]
6	60	Female	3 wk	N/D	N/D	N/D	Right colon	N/D	Segmental resection, OP	NED, 9 mo	[1]
7	35	Male	4 wk	N/D	N/D	N/D	SC	N/D	Segmental resection, OP	NED, 12 mo	[1]
8	24	Male	N/D	N/D	N/D	N/D	Jejunum	N/D	Segmental resection, OP	NED, 13 mo	[2]
9	65	Female	N/D	N/D	N/D	N/D	Jejunum	N/D	Segmental resection, OP	NED, 15 mo	[2]
10	76	Female	N/D	N/D	N/D	N/D	Jejunum	N/D	Segmental resection, OP	NED, 14 mo	[2]
11	46	Male	N/D	N/D	N/D	N/D	Colon	N/D	Segmental resection, OP	NED, 16 mo	[2]
12	49	Male	N/D	N/D	N/D	N/D	Jejunum	N/D	Segmental resection, OP	NED, 10 mo	[2]
13	59	Male	N/D	N/D	N/D	N/D	Jejunum	N/D	Segmental resection, OP	NED, 8 mo	[2]
14	39	Male	N/D	N/D	N/D	N/D	Colon	N/D	Segmental resection, OP	NED, 11 mo	[2]
15	68	Male	N/D	N/D	N/D	N/D	Gallbladder	N/D	Segmental resection, OP	NED, 17 mo	[2]
16	61	Male	N/D	N/D	N/D	N/D	Omentum	N/D	excisional biopsies of omentum	NED, 8 mo	[2]
17	42	Female	N/D	N/D	N/D	N/D	Jejunum and Colon	N/D	Segmental resection, OP	NED, 8 mo	[2]
18	72	Female	5 days	Cholelithiasis, chronic pancreatitis	None	Yes	Jejunum	N/D	Segmental resection, OP	NED, 18 mo	[3]
19	75	Female	6 days	Hypertension, rhematoid arthritis, breast carcinoma	Naproxen, lorazepam, tamoxifen, co-amlofruse	Yes	Ileum	N/D	Segmental resection, OP	Died 2 wk after discharge, recurrent mestenteric ischemia	[3]
20	31	Female	1 day	None	Oral contraceptive pill	None	Cecum and appendix	N/D	Segmental resection, OP	NED, 10 mo	[3]
21	68	Male	3 wk	Osteoarthritis, hypertension	None	Yes	Jejunum	N/D	Segmental resection, OP	NED, 6 mo	[3]
22	53	Male	3 days	None	None	Yes	Ileum	N/D	Segmental resection, OP	NED, 8 mo	[3]
23	32	Male	N/D	Ulcerative colitis	5-aminosalicylic acid	N/D	From TC to rectum	N/D	Subtotal colectomy and ileostomy, OP	NED, 5 mo	[4]
24	34	Male	3 mo	Hypertension, gout	Allopurinol	Yes	TC, SC	N/D	Total colectomy, LAP	N/D	[5]
25	65	Male	several mo	Coronary artery disease, hyperlipidemia	Clopidogrel, anlodipine, valasartan, metoprolol, atorvastatin, Asacol	N/D	From TC to rectum	N/D	Abdominoperineal resection, LAP	N/D	[5]
26	52	Male	4 mo	Glaucoma	None	N/D	SC	N/D	Hartmann’s, OP	NED, 6 mo	[6]
27	54	Female	3 mo	None	None	None	SC	N/D	Segmental resection, OP	NED, 12 mo	[7]
28	64	Female	N/D	Arthrosis	Indomethacin	Yes	Ileum	N/D	Right hemicolectomy, OP	Recurrent but alive	[8]
29	65	Male	15 days	Pyelonephritis	N/D	N/D	SC	30 days	Hartmann’s, OP	NED, 12 mo	[9]
30	39	Male	6 days	Urinary stones	None	N/D	Ileum	6 days	Segmental resection, OP	NED, 15 mo	[10]
31	24	Male	2 days	None	None	N/D	Jejunum	2 days	Segmental resection, OP	NED, 13 mo	[11]
32	32	Male	5 days	None	None	N/D	TC, DC	12 days	Subtotal colectomy, OP	NED, 24 mo	[12]
33	29	Male	10 days	None	None	N/D	Ileum	N/D	Segmental resection, OP	NED, 24 mo	[13]
34	65	Male	57 days	Depression, hypertension, prostatic hypertrophy, hyperuricemia, headache	Clonazepam, paroxetine, azilsartan, suvorexant, silodosin, tadalafil, allopurinol, tramadol hydrochloride, acetaminophen, vonoprazan fumarate	Yes	From DC to rectum	57 days	1st: Transverse colostomy, OP. 2nd: Subtotal colectomy, ileostomy, OP	NED, 17 mo	Ours

*mo* month(s), *wk* week(s), *N/D* no description, *TC* transverse colon, *DC* descending colon, *SC* sigmoid colon, *OP* open surgery, *LAP* laparoscopic surgery, *NED* no evidence of disease, *ARDS* acute respiratory distress syndrome, *DIC* disseminated intravascular coagulation

MIVOD is an extremely rare disorder, with the distinctive feature of vasculitis of mesenteric veins and their intramural tributaries. Vascular inflammation is described as either predominantly lymphocytic or neutrophilic. In some MIVOD cases, myointimal hyperplasia in mesenteric veins is evident [2]; these changes were not believed to be drug-related, because no supportive history was present and there was a lack of arterial and skin involvement. The differential diagnoses of MIVOD are as follows: superior mesenteric artery (SMA) embolism, SMA thrombosis, vasculitis (Behçet’s disease, systemic lupus erythematosus [SLE], Crohn’s disease), and mesenteric venous thrombosis [10]. Blood vessels in unaffected bowel segments are normal.

The clinical presentation of MIVOD consists of abdominal pain, nausea, vomiting, and diarrhea or bloody diarrhea with a duration of several days to months [3]. The severity of abdominal pain is out of proportion to the morphologic findings. Blood tests do not have any diagnostic significance in MIVOD. The chronic ischemic changes may result in colonic strictures, as in our patient’s case. Medical treatment is ineffective [12]. The etiology of MIVOD is incompletely understood, but case reports implicated the antiphospholipid syndrome in one patient with subsequent deep vein thrombosis (DVT) [11] and another with a cytomegalovirus (CMV) infection [13].

The diagnosis of MIVOD can only be confirmed based on a histological examination of a resected specimen, which means that the histological diagnosis can be made only following surgery [6]. It is very difficult to solve the problem of what kind of MIVOD cases need to be operated, because it is almost impossible to diagnose without resection of affected bowel. If a patient could be diagnosed as MIVOD before surgery, the timing of operation would be better after improving the edema of mesentery. But how to improve the edema of mesentery by conservative therapy has no clear answer at the moment.

There is often a delay in diagnosis. As shown in Table 1, the prognoses after surgical resections were reported to be good [3, 4, 6]. It is unclear why the pathological process is localized and why recurrence of MIVOD is seldom observed [6].

## Conclusions

We presented a case of successful surgical management for MIVOD and summarized the cases reported to date. The diagnosis of MIVOD is very difficult because it can be made only after surgery for the ischemic area of the intestine or mesentery, and some cases have shown a long time from the manifestation of the symptoms to surgery. Clinicians should consider MIVOD when examining a patient with intestinal ischemia. When MIVOD is suspected, the patient is indicated for surgery with an accurate diagnosis and good prognosis.

## Data Availability

The authors declare that all the data in this article are available within the article.

## Abbreviations

CMV: Cytomegalovirus
DVT: Deep vein thrombosis
MIVOD: Mesenteric inflammatory veno-occlusive disease
